# Epidemiological investigation and diagnostic analysis of osteonecrosis of the femoral head in three northeastern provinces of China

**DOI:** 10.1186/s13018-024-04768-y

**Published:** 2024-05-12

**Authors:** Wangyan Liu, Ju’an Yue, Xiaozhong Guo, Randong Wang, Hao Fu

**Affiliations:** Department of Joint Surgery, Aviation General Hospital, Courtyard 3, Anwai Beiyuan, Chaoyang District, Beijing, China

**Keywords:** Osteonecrosis, Femoral head, Single centre, Misdiagnosis, Lumbar disc herniation

## Abstract

**Background:**

In this retrospective case investigation, we analysed the data of patients with osteonecrosis of the femoral head (ONFH) to reveal demographic and clinical diagnostic features of ONFH in three northeastern provinces of China and provide a reference for its prevention, diagnosis, and treatment.

**Methods:**

We collected data from patients in Beijing Orthopaedic Hospital of Liaoning, focusing on the aetiology and diagnosis of ONFH. Medical records and self-designed questionnaires were used to collect information for statistical analysis, including age, aetiology, reason for glucocorticoid use, hospital level at first visit, and diagnosis.

**Results:**

In total, 906 patients with complete medical records were included in the analysis. The mean patient age was 47.65 ± 12.12 years. The peak age distribution was in the 40s for men and the 50s for women. Among the total cohort, 72 patients (7.95%; 40 men and 32 women) had traumatic ONFH, 198 (21.85%; 131 men and 67 women) had steroid-induced ONFH, 230 (25.39%; 121 men and 109 women) had idiopathic ONFH, and 406 (44.81%; 397 men and 9 women) had alcohol-induced ONFH. Six hundred and twenty patients were diagnosed with ONFH at the first visit, while 286 patients were misdiagnosed, with a diagnosis rate of 68.43%. The diagnosis rate at the first visit in tertiary hospitals was 76.14%. The diagnosis rate at the first visit in second-class hospitals was 52.07%.ONFH was most likely to be misdiagnosed as lumbar disc herniation.

**Conclusions:**

Most patients with ONFH in three northeastern provinces of China were middle-aged, male, and had alcohol-induced ONFH. The misdiagnosis rate of ONFH at the first visit was very high, especially for misdiagnosis of lumbar disc herniation, indicating that the diagnosis of ONFH requires further improvement.

## Background

Osteonecrosis of the femoral head (ONFH) involves a series of pathological changes, such as the death of bone marrow components and bone cells, subchondral fracture, and articular surface collapse, resulting in hip pain and dysfunction owing to various causes [1–3]. At present, most studies on ONFH have focused on aetiology and treatment, and there have been few epidemiological investigations. The number of new cases of ONFH in the United States increases by 10,000 to 20,000 each year [4], and approximately 75% of patients are between 30 and 60 years old [5]. In China, the number of new ONFH cases increases by 100,000 to 200,000 each year [6]. With improvement in people’s quality of life and innovations in diagnosis and treatment technologies, these proportions continue to increase each year. However, ONFH is easily misdiagnosed or missed owing to its complex aetiology, the difficulty of detecting it in its early stages, and its high disability rate.

In this study, we conducted a retrospective case investigation among 906 patients with ONFH in three northeastern provinces of China with the aim of revealing the demographic and clinical diagnosis characteristics of ONFH in these provinces and providing a reference for the prevention, diagnosis, and treatment of ONFH.

## Materials and methods

All procedures performed in this study were in accordance with the ethical standards of the World Medical Association Declaration of Helsinki Ethical Principles for Medical Research Involving Human Subjects. The study protocol was approved by the Ethics Committee of Aviation General Hospital (No: HK2019-01-04). The ethics committee of our hospital approved this retrospective analysis.

The inclusion criteria of this study were as follows: (1) ONFH diagnosed according to the “Clinical Guidelines for the diagnosis and treatment of femoral head necrosis in Adults“ [7]; (2) patients were diagnosed according to clinical diagnosis, X-ray (orthographic position, frog position), computed tomography or magnetic resonance imaging, and had complete clinical data. The exclusion criteria were: (1) < 18 years old; (2) diagnosed with tuberculosis of the hip joint, bone tumour, acetabular dysplasia, ankylosing spondylitis, necrosis of the hip joint after infection, or femoral head lesions caused by haemophilic arthritis.

### Data collection

We collected data of hospitalised patients diagnosed with ONFH between May 2023 and November 2023 in Beijing Orthopaedic Hospital of Liaoning. The medical records were initially screened through the information access system. Key words such as “femoral head necrosis”, “femoral head aseptic necrosis”, “femoral head ischemic necrosis”, and “osteonecrosis of the femoral head” were used to screen the discharge diagnosis, and we collected patient data within the retrieval time range.

### Survey form design

We designed a survey table including: (1) general information of patients: age, sex; (2) aetiology of ONFH: (i) history of alcohol abuse (defined as those reporting an average consumption of ≥ 40 g pure alcohol per day for men and ≥ 20 g pure alcohol per day for women during the previous 12 months [8]) and (ii) steroid use (systematic steroid users defined as those with an intake of ≥ 2 g of prednisone or its equivalent within a period of 3 months [9]); (3) diagnosis: first visit hospital level, preliminary diagnosis; and (4) Association Research Circulation Osseous (ARCO) stage.

### Statistical analysis

IBM SPSS version 22.0 (IBM Corp., Armonk, NY, USA) was used for the statistical analysis. Data are expressed as mean ± standard deviation. The Wilcoxon test and Kruskal–Wallis test were used to compare age groups. Rate comparisons were performed using the Pearson chi-square test and continuity correction test. *P* < 0.05 was considered to indicate statistical significance.

## Results

The relevant data of 906 inpatients who met the inclusion criteria were retrieved from the electronic medical record inquiry system, and the data were input into the information table. Patients with ONFH comprised 689 males and 217 females. The sex ratio was 3.175:1. Among them, 299 patients had unilateral ONFH and 607 had bilateral ONFH. There were 1027 hips classified as ARCO stage II, 184 classified as ARCO stage IIIA, 189 classified as ARCO stage IIIB, and 113 classified as ARCO stage IV.

### Age distribution

**Fig. 1:**
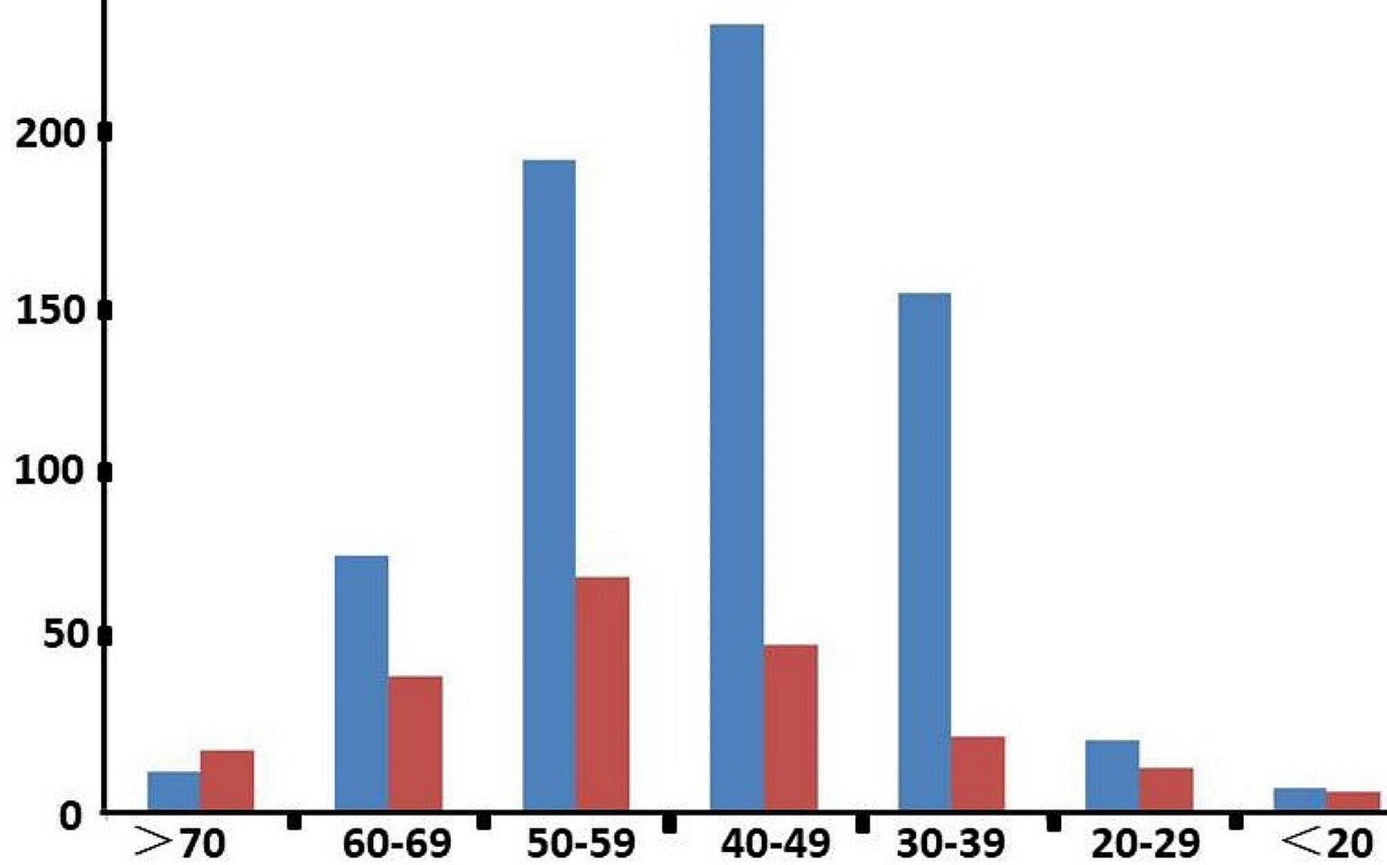
Patients with ONFH patients, by age group. Blue bars represent men and red bars women. The vertical axis shows the number of patients

The mean patient age in the total cohort was 47.65 ± 12.12 years, and the mean age was significantly older for women (51.05 ± 14.26 years) than for men (46.58 ± 11.16 years) (Z = − 5.177, *P* = 0.000). The peak age distribution was in the 40s for men and the 50s for women (Fig. 1). The average age was 48.40 ± 10.06 years among patients with alcohol-induced ONFH, 42.59 ± 13.46 years among patients with steroid-induced ONFH, 47.25 ± 14.66 years among those with trauma-induced ONFH, and 50.82 ± 12.09 among those with idiopathic ONFH. The mean age significantly differed between the four aetiological groups (χ^2^ = 44.06, *P* = 0.000). The group with the oldest age of onset was the idiopathic ONFH group, followed by the alcohol-induced, trauma-induced, and steroid-induced ONFH groups from oldest to youngest, respectively (Table 1).

**Table 1:** Age distribution of patients by sex and aetiology

Group	Age	Z/ X^2^	*P*
Gender		−5.177,	0.000
male	46.58 ± 11.16	-	-
female	51.05 ± 14.26	-	-
Aetiology		44.06	0.000
corticosteroids	42.59 ± 13.46	-	-
traumatic	47.25 ± 14.66	-	-
alcohol abuse	48.40 ± 10.06	-	-
idiopathic	50.82 ± 12.09	-	-

### Aetiological characteristics of patients with ONFH

**Fig. 2:**
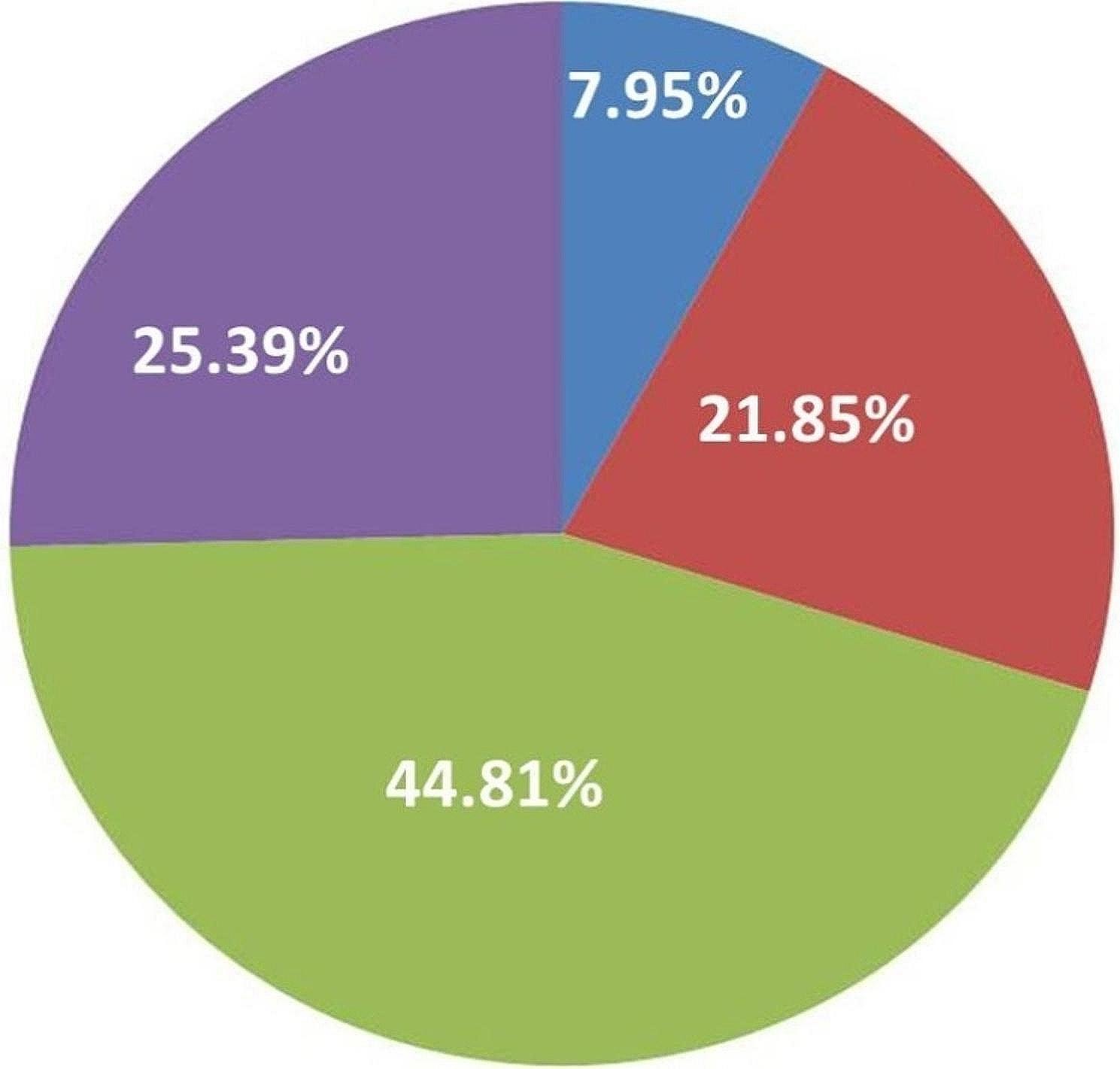
Distribution of ONFH based on four aetiologies. Green: alcohol-induced ONFH; purple: idiopathic ONFH; red: steroid-induced ONFH; blue: traumatic ONFH

**Fig. 3:**
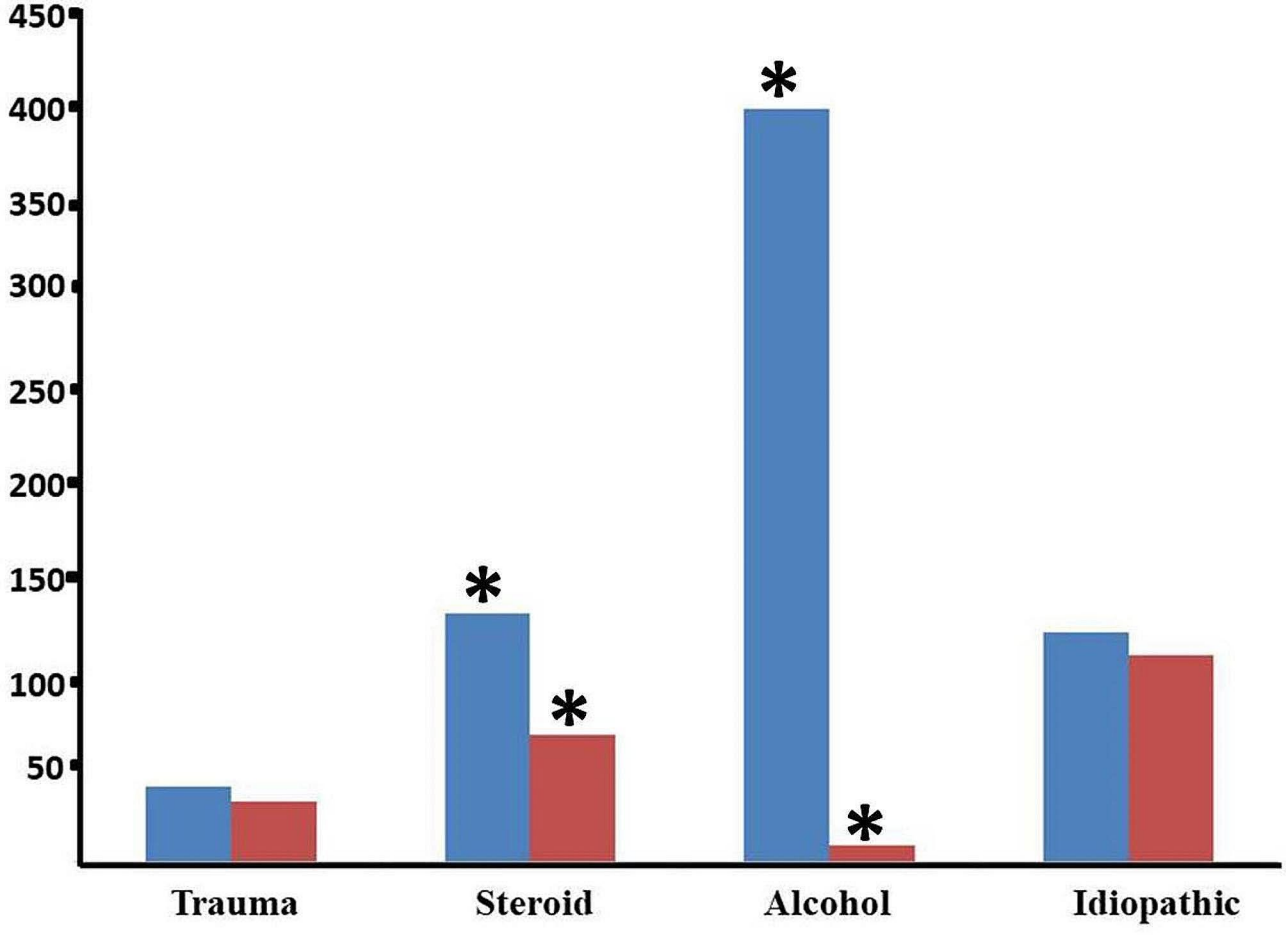
Constituent ratio of aetiologies by sex. The vertical axis shows the number of patients. Blue: males; red: females. *indicates a statistically significant difference (*P* < 0.05)

Among the total cohort (Figs. 2), 72 patients (7.95%; 40 men and 32 women) had traumatic ONFH, 198 (21.85%; 131 men and 67 women) had steroid-induced ONFH, 230 (25.39%; 121 men and 109 women) had idiopathic ONFH, and 406 (44.81%; 397 men and 9 women) had alcohol-induced ONFH. Sex was associated with different aetiologies (Fig. 3). The prevalences of steroid-induced and alcohol-induced ONFH were significantly higher in men than women (χ^2^ = 23.23, *P* = 0.000 for steroid-induced OFNH; χ^2^ = 477.87, *P* = 0.000 for alcohol-induced ONFH). Alcohol-induced ONFH was the most common type of ONFH, and mainly affected men. There were no significant differences between men and women in the prevalences of traumatic and idiopathic ONFH (*P* > 0.05).The leading three diseases that required glucocorticoid administration leading to OFNH were autoimmune disease (17%), skin disease (16.16%), and nervous system diseases (10.10%). A total of 198 patients with steroid-induced ONFH had underlying diseases (Table 2).

**Table 2:** Underlying diseases in steroid-induced osteonecrosis of the femoral head

Primary diseases	Number of Occurrences(*n*)	Ratio(%)
Autoimmune disease	46	17%
Systemic lupus erythematosus	26	-
Rheumatoid arthritis	13	-
Sicca syndrome	3	-
Adult-onset still’s disease	3	-
Behcet’s disease	1	-
Skin disease	32	16.16%
Nervous system disease	20	10.10%
Upper respiratory infection	16	8.08%
Kidney disease	11	5.56%
Allergic Rhinitis	11	5.56%
Gout	10	5.05%
Disease of the eyes	10	5.05%
Lumbago	9	4.55%
Hematological system diseases	8	4.04%
Pulmonary disease	7	3.35%
Tumour	5	2.53%
Other Disease	13	6.57%
Toothache	3	-
Vascular disease	2	-
Allergic to drug	2	-
Chronic gastritis	1	-
Subacute thyroiditis	1	-
Sudden deafness	1	-
Organ transplantation	1	-
Soft tissue infection	1	-
Otitis media	1	-

### Diagnostic analysis

**Fig. 4:**
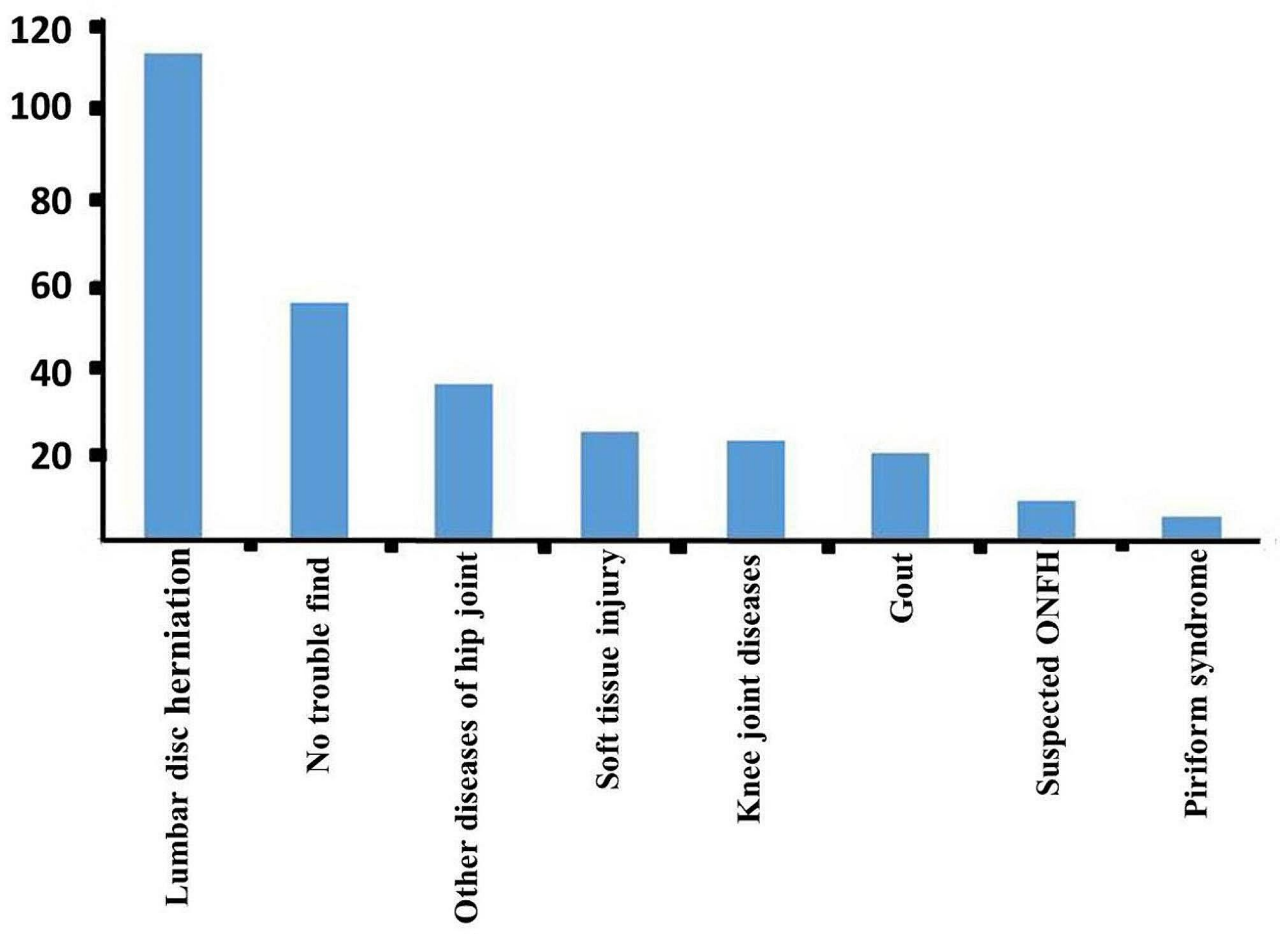
Distribution of misdiagnosed diseases. The vertical axis shows the number of patients

Of the 906 included patients, 620 patients were diagnosed with ONFH at the first visit; 286 patients were misdiagnosed, giving a diagnosis rate of 68.43% at the first visit. The diagnostic method was radiography for 226 patients, Computed tomography (CT)for 67 patients, Magnetic resonance imaging (MRI) for 261 patients, radiography plus CT for 65 patients, and radiography plus MRI for 287 patients. When misdiagnosed patients were finally diagnosed with ONFH, the proportions of patients with ARCO stages IIIA, IIIB, and IV were significantly higher than those of patients who were correctly diagnosed with ONFH at the first visit (Table 3). In the misdiagnosed group, 95 hips underwent head preservation surgery and 252 hips underwent total hip replacement; in the correctly diagnosed group, 758 hips underwent head preservation surgery and 408 hips underwent total hip arthroplasty (THA). The incidence of THA was significantly lower in the diagnosed group than in the misdiagnosed group (χ^2^ = 6.99, *P* = 0.008). Most patients with ONFH (*n* = 616) were first treated at a tertiary hospital; 469 patients were diagnosed with ONFH and 147 patients were misdiagnosed. The diagnosis rate at the first visit in tertiary hospitals was 76.14%. The first visit among 290 patients with ONFH was to a second-class hospital; 151 patients were diagnosed with ONFH and 139 patients were misdiagnosed. The diagnosis rate at the first visit in second-class hospitals was 52.07%. Misdiagnosis according to different levels of hospital is shown in Table 4, and the probability of misdiagnosis with different diseases is shown in Fig. 4. ONFH was most likely to be misdiagnosed as lumbar disc herniation.

**Table 3:** ARCO stages of patients in the two groups

Items	II (*n*/%)	IIIA (*n*/%)	IIIB (*n*/%)	IV (*n*/%)
Misdiagnosed	170/48.99	59/17.00	71/20.46	47/13.55
Diagnosed	857/73.50	125/10.72	118/10.12	66/5.66
χ^2^	22.62	9.88	26.16	24.05
P	0.000	0.002	0.000	0.000

**Table 4:** Misdiagnoses according to different level hospitals

Items	Number of patients	Diagnosis(*n*)		Diagnosis rate(%)
	(*n*)	Yes No		
Tertiary hospitals	616	469	147	66.7
Second-class hospitals	290	151	139	42.5
χ^2^/P	-	-	-	52.87/0.000

## Discussion

ONFH is a devastating disease affecting the hip joint that can cause severe clinical symptoms and greatly reduce quality of life among patients [10–14]. At present, epidemiological studies on ONFH have mainly been routine descriptive studies and aetiological studies [6, 8, 15]. In the present study, we describe in detail not only the age distribution and aetiology of ONFH but also diagnosis and misdiagnosis of OFNH according to different hospital levels.

In this study, male patients accounted for 76.05% and female patients accounted for 23.95% of the total, with a sex ratio of approximately 3.175:1, indicating that men had the highest incidence of ONFH; this finding is consistent with trends described in other studies [5, 16]. The onset time was earlier among male patients, with most developing ONFH between 40 and 49 years of age. In contrast, the peak onset age for women was 50–59 years. These patients are in the middle and younger age groups and represent the main labour force and backbone of the family and society. ONFH in these age groups seriously affects social and family life. Therefore, the prevention and treatment of ONFH should be focused on young and middle-aged populations.

The pathogenesis of ONFH is complex and remains unclear, but commonly accepted causes involve glucocorticoid use, alcohol abuse, and trauma [17–19]. In the present study, there was a very high prevalence of alcohol-induced ONFH among male patients. Connective tissue diseases remain the main reason for glucocorticoid administration [15, 20]. However, in our study, glucocorticoids were used for many conditions such as upper respiratory tract infection, allergic rhinitis, skin allergies, and muscle strain, among others. Glucocorticoids should be used with caution in patients with skin diseases, upper respiratory infection, allergic rhinitis, gout, and lumbago. Although many patients did not use glucocorticoids for long periods or in large doses [9], many had a history of using small doses at the time. The relationship between the use of low-dose hormones and the occurrence of ONFH requires further study.

A large number of patients have ONFH in China [6, 8]. The diagnosis rate of early ONFH at the first visit was only 68.43%. More than half of the patients in the study cohort presented at tertiary hospitals for their first visit, and the rate of first diagnosis of ONFH in tertiary hospitals was only 76.14%. We found that the most commonly misdiagnosed disease was lumbar disc herniation, followed by soft tissue injury. On physical examination, patients with ONFH have limited hip flexion, internal rotation, and external rotation. Additionally, MRI is up to 100% sensitive for the diagnosis of ONFH [21]. Therefore, physical examination and MRI are very valuable to avoid misdiagnosis and missed diagnosis of early ONFH. It is very important to obtain a timely diagnosis to enable the performance of ‘joint- preserving’ treatments and delay THA for as long as possible. Combining core decompression with bone grafting, bone marrow-derived cell transplantation, or other techniques seems to provide better outcomes than core decompression alone [22, 23]. However, approximately one-third of the osteotomies performed in patients with ONFH are reportedly converted to THA during a period of approximately 7 years [24]. Therefore, osteotomy should be performed cautiously in carefully selected older adult patients with ONFH [24]. The reported factors that negatively affect the postoperative efficacy of joint-preserving treatments are male sex, a longer symptom duration before treatment, a higher visual analogue scale score for pain, and a lower Harris Hip Score [25]. Joint-preserving treatments are also reportedly effective for the management of ONFH in skeletally immature patients [26].

There are some limitations in this study. First, this was a small sample size; further multi-centre research with a larger sample is required. Second, only patients diagnosed with ONFH were included in this study. Patients with hip dysplasia and primary or secondary hip osteoarthritis who were misdiagnosed with ONFH were not included. Third, further study is needed to investigate whether the severity of ONFH varies in accordance with the aetiology and patient age.

## Conclusion

Most patients with ONFH in the three northeastern provinces of China in this study were middle-aged men with alcohol-induced ONFH. The misdiagnosis rate of ONFH at the first visit was very high, especially for misdiagnosis of lumbar disc herniation, indicating that the diagnosis of ONFH requires further improvement.

## Data Availability

No datasets were generated or analysed during the current study.

## Declarations

### Abbreviations

ARCO Association Research Circulation Osseous.

Computed tomography (CT).

MRI Magnetic resonance imaging.

ONFH Osteonecrosis of the femoral head.

THA Total hip arthroplasty.

### Data and materials availability statement

All data and materials used to support the findings of this study are included within the article.
